# On Diseases of the Pancreas

**Published:** 1908-06-20

**Authors:** 

**Affiliations:** Berlin. Translation of a Paper read before the Anglo-American Medical Association, Berlin. Specially reported for The Hospital.


### HOSPITAL CLINICS.

### ON DISEASES OF THE PANCREAS.

BY DR. ALBU, Berlin.

Translation of a Paper read before the Anglo-American Medical Association, Berlin. Specially reported for THE HOSPITAL.

THE pathology of the pancreas was formerly one of the most unexplored fields of medicine. The researches of the last decade, however, have enabled us to diagnose pancreatic lesions in quite a number of cases. The difficulty of diagnosis is chiefly due to the symptomatology, which is not so distinctly pronounced as in other abdominal diseases. I shall therefore discuss all the symptoms which speak with certainty of an affection of the pancreas.

The pancreas is frequently subject to acute inflammation of its parenchyma, with inflammatory swelling leading to fatty necrosis. The diagnosis of this condition is extremely difficult, and it is often confused with the symptom-complex of intestinal obstruction. In the differential diagnosis we must keep in mind that the pancreas is situated above the umbilicus. As a rule affections of the pancreas are due to traumatism. If a patient complains of pains in this region we should always think of pancreatic disease, even if the trauma seems very insignificant. The pancreas is usually not at all considered in differentiating, and that is why acute pancreatitis is frequently not recognised.

There is a chronic pancreatitis, too, that needs consideration. Chronic pancreatitis may be parenchymatous or interstitial, and the latter may pass on into atrophy of the organ. The organ shrinks and becomes infiltrated and solid. Such atrophy is one of the commonest lesions of the pancreas, but it is not easy to distinguish between primary and secondary atrophy.

Pancreatic cysts have been considered a great deal of late years, since surgery has had a number of successful results. In addition, colica pancreatica, with intermittent attacks of pain, is to be mentioned. This may be confused with biliary colic. Finally, I wish to mention carcinoma of the pancreas, which is often a primary affection. This is a not uncommon precursor of secondary carcinoma of the liver.

The greater part of our knowledge of pancreatic diseases originates, of course, from anatomical studies, but lately the clinical features of pancreatic affections have been successfully established. I wish to emphasise here the general diagnostic symptoms. Many affections of the pancreas cause no trouble at all; for a small portion of the pancreas is sufficient for the full function of the organ, and the diagnosis is therefore clinically extremely difficult. One has even found diffuse affections of this organ *post mortem* which have caused no symptoms at all during life. In an infiltrative carcinoma of the pancreas we often find no symptoms. Hausemann explains this by a theory that the carcinoma cells are able to carry on the function of the normal cell.

We can therefore diagnose pancreatic affections only in a certain number of cases. For this purpose we have the following cardinal symptoms:—

(1) Diabetes, frequently with acetonuria. (2) Steatorrhœa. (3) Copious undigested muscle-fibres in the fæces (azotorrhœa). (4) Intermittent pancreatic colics.

These four symptoms together give the definite picture of a pancreatic disease. Diabetes by itself is not necessarily a symptom of a pancreatic affection. Although Mering and Minkowski have caused a diabetes in animals by extirpation of the pancreas, the problem is not yet solved for the human pancreas. We find, on the one hand, pancreatitis without diabetes, and, on the other, no histological alterations of the pancreas in diabetes. We have also observed diabetes in children without any visible lesion of the pancreas. Still, we must admit that the histological integrity of the pancreas is no criterion for its functional integrity. In our study of functional diseases we have learned that functional disorders may exist with an intact tissue. Just as the intestines, the heart, and the kidney, the pancreas may show a pure functional anomaly. Now we know the close relation between the ferment of the pancreas and the functions of other organs. In functional pancreatic disorder the ferment is not active and sugar comes into circulation. Therefore we have to regard pancreatic diabetes as a functional anomaly.

We come now to the second symptom, steatorrhœa. Fat is digested by the bile and the pancreatic juice, and by them is transformed into glycerine and fatty acids. If the lipolytic ferment does not act, digestion of fat ceases. Consequently the amount of lipous acids and of soaps in the fæces must be diminished, whereas the amount of neutral fat will be increased. The fat-stools appear of a light colour, grey-white. This may be noticed in pancreatic diseases even when but little fat is being ingested. Microscopically we find needles of fatty acid crystals, globules of fat, and clots of soap.

Azotorrhœa, the third symptom, is the occurrence of undigested muscle-fibres in the fæces. This is sometimes so pronounced that we recognise meat in the fæces macroscopically. Sometimes we are also able to recognise a disorder of the reabsorption of albumen. Both the metabolism of carbohydrates and that of albumen are out of order.

The fourth symptom consists of colics, similar to those occurring sometimes in diabetes. There is severe pain in the left hypochondrium, sometimes radiating into the shoulder. The pains are chiefly localised to the left side, and are easily differentiated from biliary colic. The pancreas is almost always tender to palpation. Recent researches have also furnished other symptoms, which, however, are not always reliable. Sahli has recommended glutoïd capsules containing 0.15 gr. of iodoform. The capsules are hardened by formalin. They are dissolved only in the jejunum by means of pancreatic juice. If, therefore, iodine occurs in the urine, the pancreatic juice must have dissolved the glutoïd capsules. The positive result of this experiment is decisive for the diagnosis, but not the negative. Adolf Schmidt determines the function of the pancreas by making the patient swallow small pieces of muscle-fibrils enveloped in cotton. If the nuclei of the fibrils are found in the cotton, the insufficiency of the pancreas is proved. Here the negative result of the experiment is decisive. One may also examine the function of the pancreas by administering salol. This is decomposed into phenol and salicylic acid in the intestines. This method is, however, not reliable.

By careful examinations many diseases of the pancreas have been recognised clinically within recent years. Pancreatic cysts occur most frequently. We find a fluctuating tumour in the left epigastrium behind the stomach and the transverse colon. In order to feel the tumour it is necessary to inflate the stomach. On percussion one hears a tympanitic sound. The cysts usually decrease and increase intermittently. This phenomenon should always make one suspicious of pancreatic cysts. Such cysts have also been punctured and pancreatic ferments found in them.

Pancreatic colic may be diagnosed without diabetes and steatorrhœa. It occurs almost exclusively in advanced age. In a man beyond the seventieth year with colic on the left side a suspicion of pancreatic diseases is always justified.

With therapeutics I may deal briefly. In tumours and cysts an operation is the only indication. Senn has operated on many. In pancreatic colic, as well as in acute pancreatitis, laparotomy is indicated. In pancreatic diabetes, however, in functional anomalies, and in achylia pancreatica after gastro-intestinal diseases, operation is useless. Organo-therapy has been tried in these cases. Pancreatic substances, as pankreon or pancreatin, have been administered as powder or tablets. They do not cure diabetes, but they are effective in anomalies of the pancreatic function. Fat disappears from the stools after taking pankreon, and this remedy is specific for steatorrhœa. The dose is, to begin with, three tablets per day, rising up to nine tablets, taken with meals. The digestion of fat and muscle is improved, though the application of pankreon is very limited.

In every pancreatic disease dietetic measures are of the greatest importance. Fat must be avoided entirely, or, if allowed, must be of an easily emulsified kind, as in milk and butter. The amount of meat is to be reduced; carbohydrates are to be preferred provided there is no diabetes.

